# Wearable Technologies for Detecting Burnout and Well-Being in Health Care Professionals: Scoping Review

**DOI:** 10.2196/50253

**Published:** 2024-06-25

**Authors:** Milica Barac, Samantha Scaletty, Leslie C Hassett, Ashley Stillwell, Paul E Croarkin, Mohit Chauhan, Sherry Chesak, William V Bobo, Arjun P Athreya, Liselotte N Dyrbye

**Affiliations:** 1 Department of Molecular Pharmacology and Experimental Therapeutics Mayo Clinic Rochester, MN United States; 2 Mayo Clinic Libraries Mayo Clinic Rochester, MN United States; 3 Department of Family Medicine Mayo Clinic Phoenix, AZ United States; 4 Department of Psychiatry and Psychology Mayo Clinic Rochester, MN United States; 5 Department of Psychiatry and Psychology Mayo Clinic Jacksonville, FL United States; 6 Department of Nursing Mayo Clinic Rochester, MN United States; 7 Department of Medicine University of Colorado School of Medicine Aurora, CO United States

**Keywords:** wearable, healthcare professionals, burnout, digital health, mental health

## Abstract

**Background:**

The occupational burnout epidemic is a growing issue, and in the United States, up to 60% of medical students, residents, physicians, and registered nurses experience symptoms. Wearable technologies may provide an opportunity to predict the onset of burnout and other forms of distress using physiological markers.

**Objective:**

This study aims to identify physiological biomarkers of burnout, and establish what gaps are currently present in the use of wearable technologies for burnout prediction among health care professionals (HCPs).

**Methods:**

A comprehensive search of several databases was performed on June 7, 2022. No date limits were set for the search. The databases were Ovid: MEDLINE(R), Embase, Healthstar, APA PsycInfo, Cochrane Central Register of Controlled Trials, Cochrane Database of Systematic Reviews, Web of Science Core Collection via Clarivate Analytics, Scopus via Elsevier, EBSCOhost: Academic Search Premier, CINAHL with Full Text, and Business Source Premier. Studies observing anxiety, burnout, stress, and depression using a wearable device worn by an HCP were included, with HCP defined as medical students, residents, physicians, and nurses. Bias was assessed using the Newcastle Ottawa Quality Assessment Form for Cohort Studies.

**Results:**

The initial search yielded 505 papers, from which 10 (1.95%) studies were included in this review. The majority (n=9) used wrist-worn biosensors and described observational cohort studies (n=8), with a low risk of bias. While no physiological measures were reliably associated with burnout or anxiety, step count and time in bed were associated with depressive symptoms, and heart rate and heart rate variability were associated with acute stress. Studies were limited with long-term observations (eg, ≥12 months) and large sample sizes, with limited integration of wearable data with system-level information (eg, acuity) to predict burnout. Reporting standards were also insufficient, particularly in device adherence and sampling frequency used for physiological measurements.

**Conclusions:**

With wearables offering promise for digital health assessments of human functioning, it is possible to see wearables as a frontier for predicting burnout. Future digital health studies exploring the utility of wearable technologies for burnout prediction should address the limitations of data standardization and strategies to improve adherence and inclusivity in study participation.

## Introduction

Burnout is an occupational syndrome characterized by emotional exhaustion, depersonalization, and feelings of reduced personal accomplishment caused by chronic, unmitigated high levels of job-related stress [1]. Burnout is common among health care professionals (HCPs, also referred to as health care workers), impacting an estimated 35% to 54% of nurses and physicians, and between 45% and 60% of medical students and resident physicians in the United States [2]. Several studies also reveal a high prevalence of depression and anxiety in HCPs that preceded the coronavirus pandemic [3-9]. Data further suggests that burnout and other forms of distress have increased among HCPs as a result of the COVID-19 pandemic [10-12].

This is concerning because the well-being of HCPs impacts the quality of patient care and patients’ access to care. Several meta-analyses and systematic reviews have reported associations between burnout and negative impacts on the quality of care provided to patients, including increasing the risk of medical errors [13], malpractice claims [14], nosocomial infections [15], and mortality [16]. Additionally, other studies have found that HCPs who report experiencing burnout are more likely to reduce their time taking care of patients and quit, all of which negatively impact patient’s access to care and add a burden to the global health care system [2]. The impacts of burnout go beyond the workplace, as HCPs with reported burnout are at increased risk of cardiovascular diseases [17,18], suicidal ideation [13,19], substance use disorders [20], uncontrolled stress [21], car accidents [22], and quality of life [23].

**Figure 1:**
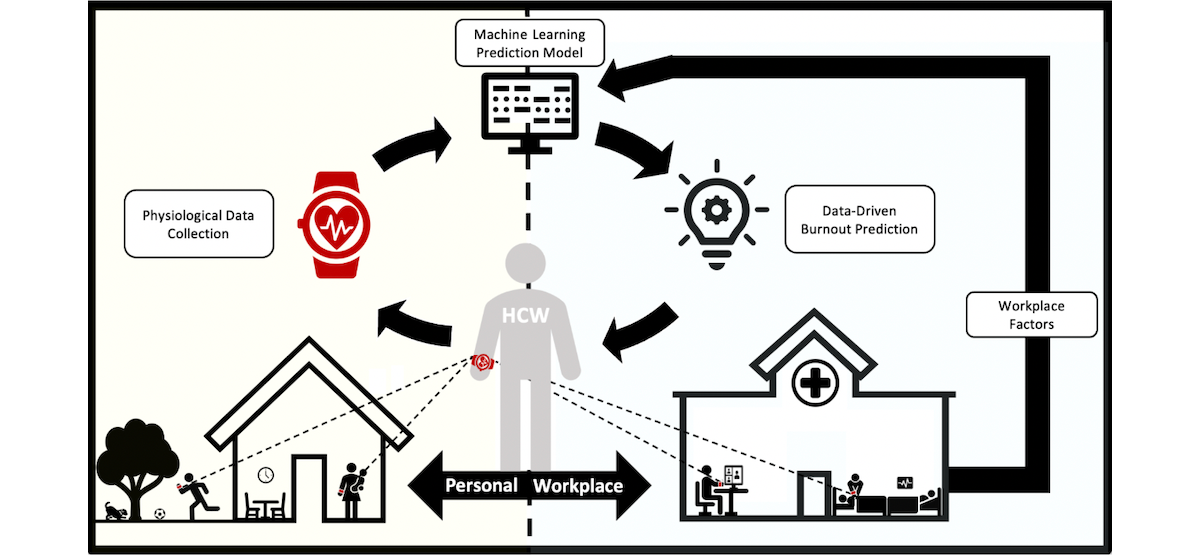
Wearable-augmented burnout management capturing the interplay of physiological and workplace factors.

Contributors of burnout in HCPs are multifactorial and complex. While most factors contributing to burnout originate from system-level factors within the work environment, some risk factors originate from the personal domain or challenges in the personal-professional interface, such as work-home conflict (Figure 1). Due to the complexity of the factors involved, no model exists for predicting when an individual HCP or group of HCPs are at risk for developing burnout or other forms of distress. In response to the negative outcomes of burnout for HCPs and patients, the National Academies of Science, Engineering, and Medicine recommends health care organizations monitor (through frequent surveys) and respond to burnout. This approach is retrospective, as the time required for health care organizations to administer surveys, HCPs to complete them, and the additional time needed to analyze and interpret results all delay any response to burnout. A better approach would be a proactive one, where organizations or individual HCPs could predict and respond to high levels of job stress before the manifestation of burnout and associated personal and professional consequences result.

Previous studies and reviews suggest heart rate (HR) [24], heart rate variability (HRV) [24], sleep [25], and skin temperature [26] vary in response to stress. Additionally, sleep or fatigue also relates to the risk of burnout [27], depression [28], and other related conditions [29]. These types of data can be collected passively from wearable devices. Over the past 5 years, the adoption of wearable devices worldwide has more than doubled [30]. Therefore, data collected passively from wearable devices could potentially provide an avenue for detecting individuals at risk for high job stress, burnout, depression, and other related conditions. If predictive, such real-time information obtained passively from wearable devices could dramatically shift the current reactive paradigm to a proactive one, potentially leading to meaningful intervention before patients and HCPs experience adverse health consequences of burnout.

Previous systematic reviews suggest wearable devices may have some utility in predicting depression severity and stress levels [31]. To our knowledge, there is no review that investigates this relationship among HCPs or explores the ability of wearable devices to detect burnout risk. Hence, a scoping review was conducted to identify and summarize studies exploring associations between burnout, anxiety, depression, and stress, with data obtained from wearable devices in cohorts of HCPs.

## Methods

### Data Sources and Search Strategy

A comprehensive search of several databases was performed on June 7, 2022. No date limits were set for the search. The databases (and their coverage periods) were Ovid: MEDLINE (1946 to Present and Epub Ahead of Print, In-Process and Other Non-Indexed Citations and Daily), Embase (1974+), Healthstar (1966+), APA PsycInfo (1987+), Cochrane Central Register of Controlled Trials (1991+), Cochrane Database of Systematic Reviews (2005+), Web of Science Core Collection via Clarivate Analytics (1975+), Scopus via Elsevier (1788+), EBSCOhost: Academic Search Premier, CINAHL with Full Text (1981+), and Business Source Premier.

The search strategy was designed and conducted by a medical librarian (LCH) with input from the study’s investigators (APA and LND). Controlled vocabulary supplemented with keywords was used. The actual strategies listing all search terms used and how they are combined are available in the Multimedia Appendix 1.

### Review Strategy

**Figure 2:**
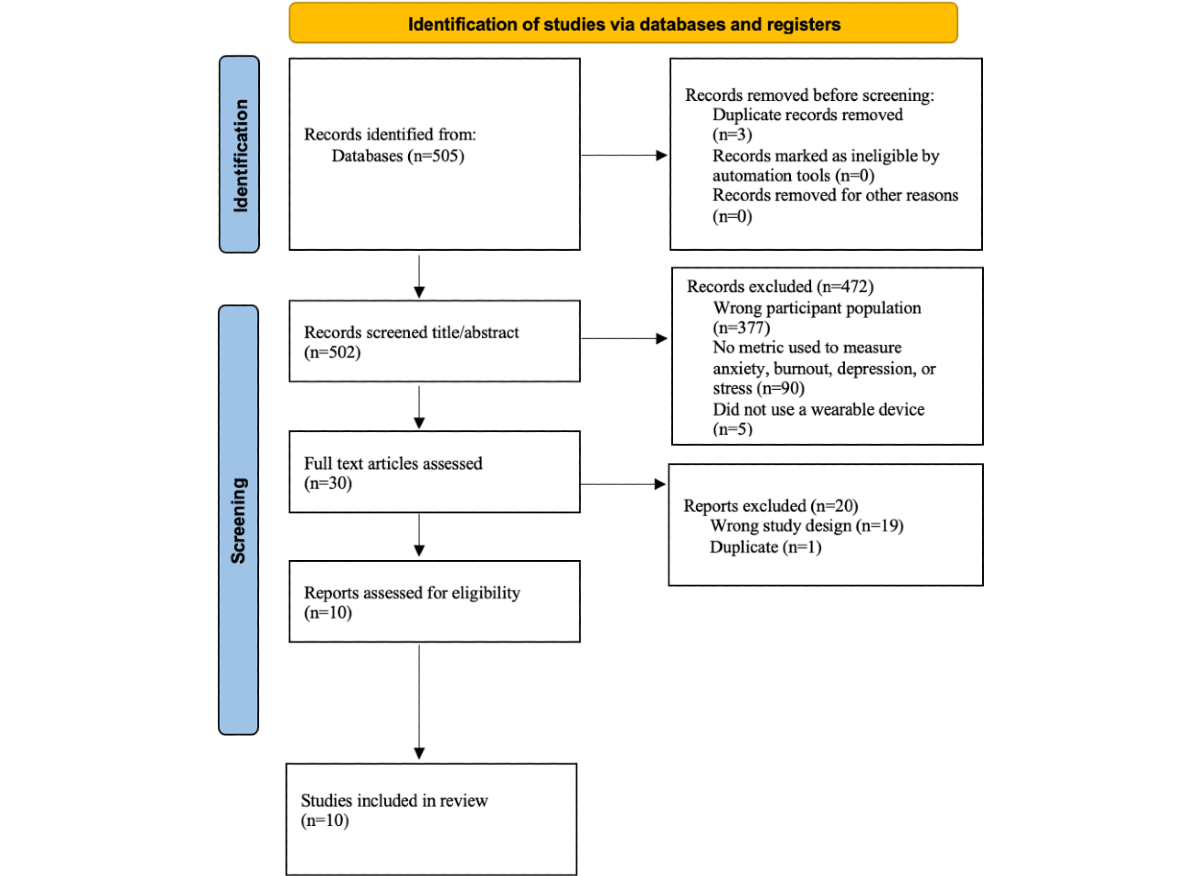
PRISMA (Preferred Reporting Items for Systematic Reviews and Meta-Analyses) diagram.

The initial search yielded 505 papers. Two reviewers (MB and SS) independently identified and screened the titles and abstracts of potentially eligible papers. The inclusion criteria of the initial round of screening were as follows: the study must include a validated measure of burnout, stress, anxiety, or depression and the study must include only data from a wearable device worn by an HCP. For this work, we defined HCP as being a medical student, resident, practicing physician, or registered nurse in a hospital or outpatient clinical setting. The full-text reviews of the papers that resulted from the initial screening, data extraction, and quality assessment were also performed independently and in pairs by 2 reviewers (MB and SS). Papers were not excluded due to their calculated quality score. During this process, 475 papers were omitted because they did not satisfy the inclusion criteria (n=472) or were duplicates (n=3). After the initial screening, the full text of 30 papers was assessed for eligibility. Any disagreement was resolved by consensus with other senior reviewers (APA and LND) and the final source list was created, with senior reviewers blinded to reviews of each other and primary reviewers (MB and SS). The study selection process is illustrated in Figure 2. Tables 1 and 2 provide descriptions of the final 10 papers published from April 2017 to December 2021 included in this review.

**Table 1:** Summary of included studies.

Author	Sample characteristics	Wearable-derived measurements	Validated anxiety, burnout, stress, or depression measures	Other measure included
Feng et al [32]	113 Nurses	HR^a^, Sleep, and STC^b^	STAI^c^	Positive and Negative Affect Schedule, Satisfaction with Life Scale, Pittsburgh Sleep Quality Index, Affect EMA^d^, Big Five Inventory-2, and Anxiety and Stress EMA^e^
Adler et al [33]	775 Residents	HR, Sleep, and STC	PHQ-9^f^	Mood EMA
Jevsevar et al [34]	21 Resident and Physicians	HRV^g^, RHR^h^, RR^i^, and Sleep	MBI-Abbreviated	—^j^
Silva et al [35]	83 Medical students (19 had complete data)	HR and HRV	PSS-4^k^	—
Mendelsohn et al [36]	59 Residents	Sleep and STC	MBI-HSS^l^	Short-Form Health Survey, Epworth Sleepiness Scale, Satisfaction with Medicine Scale, and International Physical Activity Questionnaire
Marek et al [37]	28 Residents	RHR, Sleep, and STC	Single-item burnout measure	—
Sochacki et al [38]	21 Physicians	Sleep	MBI-HSS, PROMIS-29^m^ (Depression and Anxiety)	—
Chaukos et al [39]	75 Residents (26 had complete data)	Activity level and Sleep	MBI–HSS, PSS-10, and PHQ-9	Functional Assessment of Chronic Illness Therapy-Fatigue, Penn State Worry Questionnaire, Revised Life Orientation Test, Interpersonal Reactivity Index Perspective-Taking subscale, Measure of Current Status-Part A, and Cognitive Affective Mindfulness Scale
de Looff et al [40]	114 Nurses	SC^n^	MBI–HSS (modified Dutch version)	—
Weenk et al [41]	20 Residents and Physicians	HR and HRV	STAI-short version	—

^a^HR: heart rate.

^b^STC: step count.

^c^STAI: State-Trait Anxiety Inventory.

^d^EDA: electrodermal activity.

^e^EMA: ecological momentary assessment.

^f^PHQ-9: Patient Health Questionnaire.

^g^HRV: heart rate variability.

^h^RHR: resting heart rate.

^i^RR: respiratory rate.

^j^Not available.

^k^PSS: Perceived Stress Scale.

^l^MBI-HSS: Maslach Burnout Inventory–Human Services Survey.

^m^PROMIS**:** Performance of the Patient-Reported Outcomes.

^n^SC: skin conductance.

**Table 2:** Primary findings and bias in studies using wearable devices in health care professionals.

Author	Device	Length of data collection	Primary findings	Newcastle Ottawa Scale Score
Feng et al [32]	Fitbit Charge 2	10 weeks	Baseline STAI^a^ score did not relate to sensor-measured physical activity or sleep over the ensuing 10 weeks.	8
Adler et al [33]	Fitbit Charge 2	14 months	Quarterly measurements of change in depressive symptoms related to measured STC^b^, sleep, and HR^c^.	7
Jevsevar et al [34]	WHOOP	12 weeks	Being in the operating room related to the next day HRV^d^. Device reported sleep related to next-day HRV. Relationship between baseline burnout score and device measurements not reported.	8
Silva et al [35]	Microsoft Smart Band 2	2 weeks	Stress and HRV were both significantly different between the baseline and stress condition	8
Mendelsohn et al [36]	Fitbit Charge	14 days	Baseline burnout score did not relate to average daily sleep or STC over the ensuing 14 days.	7
Marek et al [37]	Fitbit Charge HR	16 weeks	Average daily sleep and activity level over a 2-4–week period did not relate to single-item burnout measure score. Average daily resting HR over a 2-4–week period was higher among residents with burnout versus those without burnout	8
Sochacki et al [38]	WHOOP	4 weeks	No significant association between weekly burnout score and device-measured hours of sleep over 4 weeks.	8
Chaukos et al [39]	Basis Health Tracker	6 months	No association between baseline depressive symptoms or stress levels and device-measured sleep or activity levels over 30 or 90 days of the study. No association between chronic burnout (burnout at 2 time points), never burned out, new burnout (burnout at 2nd but not 1st time point), and unknown burnout status (survey not completed) and devise measured sleep or activity level aggregated over first 30 days.	6
de Looff et al [40]	Empatica E4	1 day or night shift	Skin conductance collected over 1 shift among nursing staff did not correlate with burnout scores collected on questionnaires completed within 2 days of wearing the device (mean 2.4, SD 10 days; range 0-44 days).	8
Weenk et al [41]	HealthPatch	Up to 3 days (at least 2)	Stress measured by the patch increased during surgery, more so for less experienced trainees, but did not correlate with change in STAI score before or after surgery, perhaps due to small sample size or lack of sensitivity to change.	8

^a^STAI: State-Trait Anxiety Inventory.

^b^STC: step count.

^c^HR: heart rate.

^d^HRV: heart rate variability.

### Extraction Strategy

Data extraction was mostly completed by a single researcher (MB). Other researchers (APA and SS) helped refine data extraction and review the tables. The following information was extracted from the papers and is included in Tables 1 and 2: sample population (size and occupation), anxiety, burnout, stress or depression assessment instrument, additional measurements used, wearable device used, measured physiological variable, study duration, primary findings, and the author-determined quality assessment score.

### Quality Assessment

The methodological quality of nonrandomized or observational studies was assessed by 2 reviewers (MB and SS) using the Newcastle Ottawa Quality Assessment Form for Cohort Studies [42]. The Newcastle-Ottawa Scale is a validated scale of 8 items in 3 domains: selection, comparability, and outcome. Studies are rated from 0 to 9, with those studies rating 0-2 (poor quality), 3-5 (fair quality), and 6-9 (good or high quality). All 10 studies received a Newcastle-Ottawa Scale rating of good or high quality.

## Results

### Roles of Participating Health Care Professionals

Among the 10 reviewed studies, 8 were conducted in the United States, 1 study was conducted in Portugal [35], and another one was conducted in Canada [36]. Seven studies recruited either resident physicians (postgraduate medical trainees), practicing physicians, or a combination of both, primarily within the same specialty (eg, orthopedic surgery and emergency medicine). Two studies recruited registered nurses [32,40] and 1 study recruited medical students [35]. Sample sizes ranged from 20 to 775 participants per study (see Table 1). Only 3 studies had more than 100 participants [32,33,40].

### Wearable Devices, Physiological Variables Collected, and Duration of Observation

Table 1 summarizes the sample population, sample size, physiological variables collected from wearable devices, and psychometrics used in the 10 studies. The devices used, length of data collection, and primary findings are listed in Table 2. Out of the 10 studies, 9 used wrist-worn biosensors, such as the Fitbit Charge (n=4) [32,33,35,40] WHOOP (n=2) [34,38], Basis B1 (n=1) [35], Empatica E4 (n=1) [40], and the Microsoft Smart Band 2 (n=1) [35]. Sensors embedded within wrist-worn biosensors included optical heart sensors, electrical heart sensors, accelerometers, and skin temperature sensors. The other device used was a HealthPatch, an adhesive patch with 2 ECG electrodes used to measure HR and HRV. A variety of physiological variables were collected, with sleep being the most common, measured in 7 studies. Studies ranged in length of data collection, from a single 12-hour shift to a 14-month period. Only 5 studies collected data for more than 10 weeks [32-34,37,39].

### Methodological Wearable Data Reporting

Only 2 studies explicitly stated the sampling frequency used when processing data from the wearable device [33,39]. Four of the studies discussed how the data were processed; however, the level of detail varied [32,33,35,40]. Three of the studies indicated the cutoff values for physiological variables or explained how outliers were addressed [32,33,40]. Only 4 studies explicitly stated how much raw data were retrieved from the devices [32-34,36].

### Reported Relationships Among Burnout, Depressive Symptoms, Stress, and Anxiety With Data Obtained From Wearable Devices

#### Burnout

Of the 10 included studies, 6 included a measure of burnout (Table 1) [34,36-40]. Four of these 6 studies used the Maslach Burnout Inventory–Human Services Survey (MBI-HSS) [43]. In a cross-sectional study of 114 nurses, no relationship was found between MBI-HSS score and skin conductance, a measure of autonomic nervous activity, collected through an Empatica E4, for 1 shift [40]. Another study investigated the relationship between MBI-HSS score, self-reported work hours, physical activity, and sleep, as measured by a Fitbit, in a cohort of 59 residents [36]. No relationship was found between the change in burnout score and data collected from the Fitbit over 2 weeks. In the third study, no relationship was found between MBI-HSS score and sleep, as measured by a WHOOP, over the course of 4 weeks [38]. Last, in a study of 75 medicine and psychiatry residents, no relationship was found between burnout score and sleep or activity levels, as measured by Basis B1 health-tracking device, during their first 6 months of residency [39].

Two studies measured burnout using scales other than the 22-item MBI-HSS (widely considered the gold standard) [34,37]. In a study of 21 orthopedic residents and surgeons, no association was found between baseline abbreviated MBI scores and WHOOP measures collected over 12 weeks [34]. The final study investigated the association between burnout, as measured by a commonly used single-item measure, and sleep and activity level, as measured by a Fitbit. In this study, of 28 emergency medicine residents, there was no association between burnout scores and sleep or activity levels over the course of the 16-week study [37].

#### Depressive Symptoms, Stress, and Anxiety

A 14-month study of 775 medical residents found a relationship between depressive symptoms, as measured by the 9-item Patient Health Questionnaire [44], and step count (STC) and sleep as measured by a Fitbit Charge 2 [33]. Medical residents whose depressive symptoms worsened over the period of the study had a significantly higher skew in their hourly STC distributions and spent less time in bed than those whose symptoms did not worsen. In a study of 83 medical students, Perceived Stress Scale-4 scores related to HR and HRV, were measured by a Microsoft Smartband 2, at baseline and during an examination [35].

In a 10-week study of 113 nurses led by Feng et al [32], no relationship was found between the level of anxiety, as measured by the State-Trait Anxiety Inventory (STAI) [45], and wearable sensor data (eg, sleep and HR) collected using Fitbit Charge 2 smartwatch. Weenk et al [41] conducted a study of 20 surgeons and surgical residents who completed an abbreviated version of the STAI before and after performing surgery, and wore a HealthPatch. This adhesive patch calculates stress using an HR and HRV-dependent algorithm for 48 to 72 hours [41]. There was no correlation found between the STAI score and HealthPatch data.

### Device Use Compliance and Experience

Seven studies reported data on participant adherence or experience with wearable devices. Chaukos et al [39] reported that 25 (40%) of their participants wore their device for more than 50% of the time for the first 3 months of the study, while another 13 (21%) participants wore the device for more than 75% of the time for the first 3 months. Other studies, such as one conducted by Sochacki et al [38] reported that of the 26 participants, 5 did not complete the minimum WHOOP compliance (4 weeks). Surgeons involved in a study by Jevsevar et al [34] reported a high percentage of device compliance at 83.2% of the total collection window, similar to the 93% compliance rate reported by Mendelsohn et al [36] and Sochacki et al [38]. Weenk et al [41] reported that 6 of 20 individuals experienced problems with their HealthPatch, similar to Marek et al [37] who reported 1 of 30 participants dropped out due to fitness tracker intolerance. Problems included connection failure (n=2), loss of skin contact (n=2), and skin irritation (n=2). Feng et al [32] noted similar compliance between day-shift participants and night-shift participants (number of recordings day-shift: mean 44.6, SD 3.1 sessions; night-shift: mean 45, SD 20.2 sessions).

### Risk of Bias

**Figure 3:**
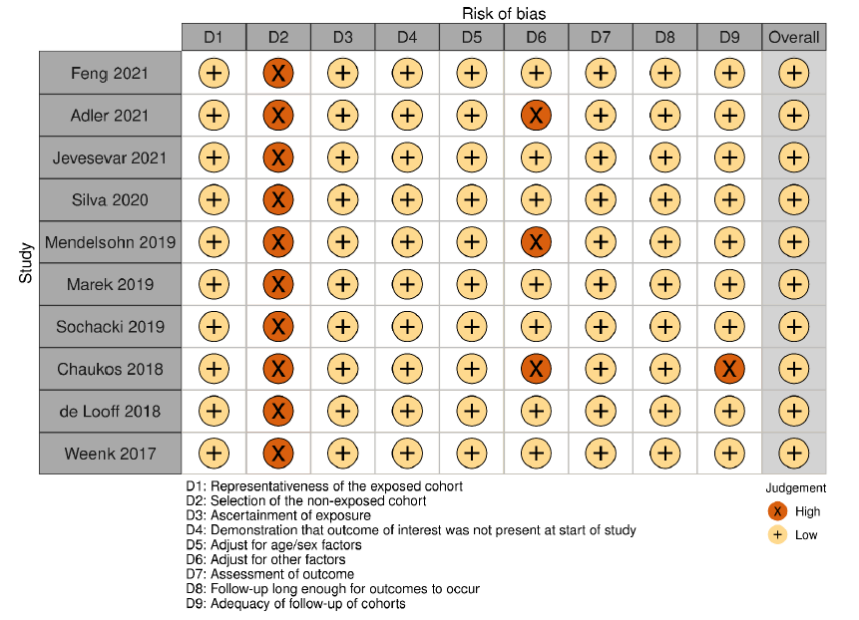
Risk of bias assessment for observational cohort studies.

A risk of bias of assessment was completed for the 8 cohort studies and 1 cross-sectional study (Figure 3). While the risk of bias was generally low across the studies, none included a comparison group of participants who did not wear a device.

## Discussion

To our knowledge, this is the first scoping review to investigate the use of wearable technologies for the prediction of burnout, anxiety, depression, and stress in HCPs. Among the 10 studies identified, a range of wearables collected data on HR, HRV, respiratory rate, skin temperature, sleep, and activity levels from a single shift of work and up to 14 months of data collection in relatively small samples of physicians, medical students, and nurses. In these studies, no relationships were found between collected physiological data from wearables and burnout or anxiety. One study reported a relationship between STC, time in bed, and depressive symptoms, and another between HR, HRV, and acute stress (during an examination). Identified studies had methodological limitations, including short duration which limits the capture of naturalistic variations in the workplace stressors.

In this review, 3 studies measured HRV [34,35,41] and only 1 found a significant relationship between HRV and acute stress. A previous systematic review involving non-HCPs identified 2 studies demonstrating relationships between HRV and acute stress-induced conditions and 1 study demonstrating a relationship between HRV and stress levels measured by catecholamine levels [31]. This previous systematic review also identified 1 study where in a setting of laboratory-induced stress, HRV parameters related to STAI score. These studies, however, differed substantially from the ones included in this review. For example, none of them collected physiological data longer than 24 minutes, stress was induced in a laboratory setting (vs occurring naturally in a work setting), and only 1 study compared physiological data with a self-reported stress measure (ie, STAI score).

Given these early findings, further research focusing on the following elements of rigor are warranted. First, the length of observation should be long enough (at least 2 or 3 consecutive quarters of a calendar year) to allow sufficient quanta of wearable data to capture fluctuations in and chronicity of workplace stress. Studies should systematically collect data using validated instruments measuring burnout (eg, MBI-HSS [43]), depression (eg, Center for Epidemiologic Studies Depression Scale [46] and Patient Health Questionnaire-9 [44]), and anxiety (eg, General Anxiety Disorder-7 [47]). Investigators may also want to consider designing cohorts comprising groups of HCPs defined by their type of medical specialty or practice location. For example, it is possible that workplace stressors, patient acuity, and job demand fluctuate between primary care and surgical specialties and between outpatient practices and hospital-based practices. Hence, the burnout biomarkers may vary between practices. Considering that burnout is defined as when job demands exceed job resources, it is possible that the workplace (eg, patient acuity and hospital bed size) and related staffing factors (eg, workload, shift length, and availability of support staff) impact physiological biomarkers collected from wearables. Hence, future studies should consider collecting organizational variables to better understand the systemic contributors of burnout. Additionally, given the era of decentralized health care practice (eg, nontraditional shift days/hours and remote care with augmented reality), studies engaging with HCPs may benefit from no-contact passive monitoring and a digital app interface for survey collection (ie, decentralized trail). Finally, there is a bioethics component to understand how wearables can be successfully integrated into workforces’ burnout management. Greater attention needs to be paid to participant engagement, including addressing comfort with wearing the device, resolving discrepancies in wearable-derived data versus self-reported data, and understanding factors that influence perceptions of fatigue but not recorded sleep [37,48,49].

The use of wearables to detect the functioning states of human beings is an active and rapidly evolving field. Several wearable-based studies have been shown to aid in the detection of mental health conditions or resilience in quality of life [50] through mindfulness practices including physical activity [51] and sleep [52-54] monitoring. Prior work has demonstrated that aspects of physical functioning when combined with data during the day could predict variations in aspects of QoL and mental well-being [55-58]. Work by Campbell et al [59-64] has demonstrated the ability of daily journaling, wearables, and mobile assessments to detect depressive symptoms and mental states in patients with schizophrenia. These prior efforts in the field of mental health and the work summarized in this scoping review demonstrate the promise of wearables in predicting states of one’s functioning, including burnout. However, a consensus is lacking on the best approaches to collecting, processing, and reporting physiological data, much like CONSORT (Consolidated Standards of Reporting Trials) [65] for reporting randomized trials and STROBE (Strengthening the Reporting of Observational Studies in Epidemiology) [66] guidelines for reporting observational studies. Standardization of variables should include the creation of a guideline for reporting the sampling frequency, device adherence, and other information regarding device parameters that impact data collection. Such standardization would assist with generalizing findings, validating predictive algorithms, informing meta-analysis, and the use of data for retraining predictive models regardless of the wearable’s make and model. Additionally, there needs to be consensus around approaches to address bioethics, privacy, and confidentiality concerns of participants [67,68]. Predictive technologies, informed by personal biometric or physiologic data, may help improve work conditions but could also place individuals’ privacy or perhaps even their job security at risk.

This study has limitations. Only studies that included physicians, resident physicians, medical students, and nurses and were published in English were included. Following the 2019 pandemic, physicians identifying as 2 or more races experienced the highest levels of burnout onset, according to a report by the American Medical Association [69]. Furthermore, there are known disparities in the access to, and the use of digital health technologies in underrepresented minorities [70,71]. Therefore, it is vital to understand the factors that cause burnout in these groups of professionals and remove barriers to access to personalized wellness technologies using wearables that may help understand and mitigate burnout. In the context of the use and access of digital health for burnout, 8 of the 10 studies reported the gender breakdown of participants, and only 1 study reported the race of their participants. With the urgent need to broaden access to digital health solutions to study and understand burnout, future efforts should (1) follow reporting guidelines (eg, set by National Institutes of Health in the Human Subjects sections) to report on participant characteristics by ethnicity, race, and gender, and (2) innovate study procedures (eg, decentralized protocols) that improve the recruitment and engagement of underrepresented minorities in digital health studies of burnout. Although we sought to include validated measures of burnout, stress, depression, and anxiety, the instruments used in the studies varied in their psychometric strengths. Finally, most studies lacked power calculations, making findings, effect sizes, or impact of dropouts difficult to interpret from the perspective of the generalizability of biomarkers.

Despite the popularity of wearable devices, only 10 studies were identified that explored relationships between physiological data and burnout, depressive symptoms, stress, or anxiety. Most of these studies had substantial methodological limitations, and nearly all reported limited data collection and processing information, participant experience with the wearable device, and device compliance. Standardizing study procedures, common data elements, and reporting of wearable data are needed to strengthen the rigor of digital health studies. Addressing these limitations will result in improvements in wearable device research, including data standardization and reporting, that will validate their use in providing early intervention for HCP wellness. Additional research is warranted to explore the potential of wearable devices, perhaps augmented with other system-level data (eg, work shift lengths and absenteeism), to predict burnout and other forms of distress, hopefully leading to meaningful action before it has an adverse impact on HCPs and patient care.

## Appendix

Multimedia Appendix 1 Search strategy.

Multimedia Appendix 2 PRISMA-ScR checklist.

## Abbreviations

CONSORT: Consolidated Standards of Reporting Trials
HCP: health care professional
HR: heart rate
HRV: heart rate variability
MBI-HSS: Maslach Burnout Inventory–Human Services Survey
STAI: State-Trait Anxiety Inventory
STC: step count
STROBE: Strengthening the Reporting of Observational Studies in Epidemiology
