# Supplementary material for: Wearable Technologies for Detecting Burnout and Well-Being in Health Care Professionals: Scoping Review
Source: J Med Internet Res. 2024 Jun 25;26:e50253. doi: 10.2196/50253 (PMC11234055; doi:10.2196/50253)
Supplement: Multimedia Appendix 1 [file jmir_v26i1e50253_app1.docx]

**SUPPLIMENTAL MATERIAL LEGENDS**

**Appendix 1.** Actual Search Strategies

**TABLE LEGENDS**

**Table S1.** Summary of included studies

**Table S2.** Outcomes measured across included studies

**FIGURE LEGENDS**

**Figure S1.** Wearable-augmented burnout management. Factors contributing to burnout originate from system-level factors within the work environment, and some risk factors originate from the personal domain or challenges in the personal-professional interface, such as work-home conflict. Real time information obtained passively from wearable devices could dramatically shift the current reactive paradigm to a proactive one, leading to meaningful intervention before patients and HCPs suffer.

**Figure S2.** PRISMA diagram

**Figure S3.** Risk of bias assessment

**SUPPLIMENTAL MATERIAL**

**Appendix 1. Actual Search Strategies**

**Ovid**

Database(s): **Embase**1988 to 2022 Week 22**, Ovid Healthstar**1966 to April 2022**, Ovid MEDLINE(R) and Epub Ahead of Print, In-Process, In-Data-Review & Other Non-Indexed Citations**1996 to June 06, 2022**, APA PsycInfo**1987 to May Week 5 2022**, EBM Reviews - Cochrane Central Register of Controlled Trials**May 2022**, EBM Reviews - Cochrane Database of Systematic Reviews**2005 to June 1, 2022
Search Strategy:

| **#** | **Searches** |
| --- | --- |
| 1 | exp Health Personnel/ |
| 2 | exp Physicians/ |
| 3 | Nurses/ |
| 4 | (physician* or doctor* or interns or resident or residents or residency or nurse or nurses or clinician*).ti. |
| 5 | ((house or attending or medical or hospital or healthcare or "health care" or clinical or "acute care") adj2 (staff or personnel or professionals or officer* or workers or employee*)).ti. |
| 6 | or/1-5 |
| 7 | Wearable Electronic Devices/ or exp wearable computer/ |
| 8 | (wearable* or "smart watch*" or smartwatch* or "apple watch*" or "smart glass*" or smartglass* or "google glass*" or "fitness tracker*" or "activity tracker*" or Garmin* or Fitibit* or Oura).ti,ab,hw,kf,tw. |
| 9 | *Biosensing Techniques/is [Instrumentation] |
| 10 | *Monitoring, Physiologic/is |
| 11 | (sensor* or biosens*).ti,ab,kf. |
| 12 | or/9-11 |
| 13 | 12 and wear*.ti,ab,kf,tw. |
| 14 | 7 or 8 or 13 |
| 15 | 6 and 14 |
| 16 | burnout, professional/ or occupational stress/ or "quality of work life"/ or work related illnesses/ or Stress, Psychological/ or fatigue/ or anxiety disorders/ or depressive disorders/ or quality of life/ |
| 17 | ((burn* adj out) or burnout* or fatigue* or resilienc* or mindfulness* or "work-life" or workload or "sick leave" or absentee* or "emotional exhaustion" or "physical exhaust*" or (quality adj1 life) or wellbeing or "well-being" or wellness).ti,ab,hw,kf,tw. |
| 18 | 16 or 17 |
| 19 | 15 and 18 |
| 20 | remove duplicates from 19 |

**SCOPUS via Elsevier 1788 +**( ( ( TITLE-ABS-KEY ( physician*  OR  doctor*  OR  nurse  OR  nurses )  OR  TITLE-ABS-KEY ( ( medical  OR  hospital  OR  healthcare  OR  "health care" )  W/1  ( staff  OR  personnel  OR  officer*  OR  workers  OR  employee* ) ) )  AND  ( TITLE-ABS-KEY ( wearable*  OR  "smart watch*"  OR  smartwatch*  OR  "apple watch*"  OR  "smart glass*"  OR  smartglass*  OR  "google glass*"  OR  "fitness tracker*"  OR  "activity tracker*"  OR  garmin*  OR  fitibit*  OR  oura ) )  AND  ( TITLE-ABS-KEY ( ( burn*  W/1  out )  OR  burnout*  OR  fatigue*  OR  resilienc*  OR  mindfulness*  OR  "work-life"  OR  workload  OR  "sick leave"  OR  absentee*  OR  "emotional exhaustion"  OR  "physical exhaust*"  OR  ( quality  W/1  life )  OR  wellbeing  OR  "well-being"  OR  wellness ) ) )  AND NOT  ( INDEX ( embase )  OR  INDEX ( medline )  OR  PMID ( 0*  OR  1*  OR  2*  OR  3*  OR  4*  OR  5*  OR  6*  OR  7*  OR  8*  OR  9* ) ) )  AND NOT  ( TITLE ( patients  OR  "patient care" ) )  AND  ( LIMIT-TO ( DOCTYPE ,  "ar" ) )

**Web of Science Core Collection via Clarivate Analytics (1975+)**

| 1 | (TS=(physician* or doctor* or nurse or nurses) or TI=((medical or hospital or healthcare or "health care") NEAR/1 (staff or personnel or officer* or workers or employee* or professionals))) and TS=(wearable* or "smart watch*" or smartwatch* or "apple watch*" or "smart glass*" or smartglass* or "google glass*" or "fitness tracker*" or "activity tracker*" or Garmin* or Fitibit* or Oura) |
| --- | --- |
| 2 | TI=(burnout* or fatigue* or resilienc* or mindfulness* or "work-life" or workload or "sick leave" or absentee* or "emotional exhaustion" or "physical exhaust*" or wellbeing or "well-being" or wellness) |
| 3 | 1 and 3 |
| 4 | TI=( patients OR "patient care" ) |
| 5 | 3 not 4 |
| 6 | PMID=(0* or 1* or 2* or 3* or 4* or 5* or 6* or 7* or 8* or 9*) |
| 7 | 5 not 6 |
| 8 | 7 Refined By:Document Types: Articles |

**EBSCOhost**
CINAHL with Full Text (1981+), Business Source Premier (1998+), Academic Search Premier

| 1 | (physician* or doctor* or nurse or nurses) or TI ((medical or hospital or healthcare or "health care") N1 (staff or personnel or officer* or workers or employee* or professionals)) |
| --- | --- |
| 2 | TI (burnout* or fatigue* or resilienc* or mindfulness* or "work-life" or workload or "sick leave" or absentee* or "emotional exhaustion" or "physical exhaust*" or wellbeing or "well-being" or wellness) |
| 3 | [TI](https://web.s.ebscohost.com/ehost/history?vid=26&sid=b0e9daa0-9f7e-4ac8-947a-1fda44c463b6%40redis) (wearable* or "smart watch*" or smartwatch* or "apple watch*" or "smart glass*" or smartglass* or "google glass*" or "fitness tracker*" or "activity tracker*" or Garmin* or Fitibit* or Oura) |
| 4 | 1 and 2 and 3 |
| 5 | TI ( patients OR "patient care" ) |
